# A Comprehensive Review on Separation Methods and Techniques for Single-Walled Carbon Nanotubes

**DOI:** 10.3390/ma3073818

**Published:** 2010-06-30

**Authors:** Naoki Komatsu, Feng Wang

**Affiliations:** Department of Chemistry, Shiga University of Medical Science, Seta, Otsu, Shiga 520-2192, Japan; E-Mail: psfwang@belle.shiga-med.ac.jp (F.W.)

**Keywords:** carbon nanotubes, separation

## Abstract

Structural control of single-walled carbon nanotubes (SWNTs) is attracting enormous interest in view of their applications to nanoelectronics and nanooptics. Actually, more than 200 papers regarding separation of SWNTs have been published since 1998. In this review, they are classified into the following five sections according to the separation methods; electrophoresis, centrifugation, chromatography, selective solubilization and selective reaction. In each method, all literature is summarized in tables showing the separated objects (metallic/semiconducting (M/S), length, diameter, (*n*, *m*) structure and/or handedness), the production process of the used SWNTs (CoMoCAT, HiPco, arc discharge and/or laser vaporization) and the employed chemicals, such as detergents and polymers. Changes in annual number of publications related to this subject are also discussed.

## 1. Introduction

Structural control of single-walled carbon nanotubes (SWNTs) is attracting enormous interest because the physical properties are closely correlated to their structures and the application frequently requires homogeneous properties. In this context, extensive investigations have been made in pursuit of selective synthesis and separation of SWNTs with specific structural properties. As for the synthesis, limited (*n*, *m*) structures of SWNTs were synthesized using a silica-supported Co-Mo catalyst [1,2] and the catalyst system of Co-incorporated mobile composition of matter 41 (Co-MCM-41) [3,4]. Great progress has been made quite recently on the synthesis using non-metal catalysts [5,6,7,8,9], and selective synthesis of metallic and semiconducting SWNTs [10,11]. 

On the other hand, separation of SWNTs is an alternative way to obtain SWNTs with specific electrical and structural properties, and a wide variety of methods have been devised so far for the separation according to metallic and semiconducting (M/S) property, diameter, length, roll-up index ((*n*, *m*) structure) and handedness (*M* or *P* structure defined in Figure 2 and Figure 3). Herein, papers related with CNT separation are categorized and tabulated according to the five methods based on electrophoresis, centrifugation, chromatography, selective solubilization and selective reaction. As for solubilization of carbon nanotubes (CNTs), there have been a number of reviews [12,13,14,15,16,17]. Several excellent reviews have also been published on separation of CNTs [18,19,20,21,22,23,24]. Therefore, the authors mainly focus on the table summary of the papers reported so far and make a brief overview in each section. On the basis of the data summarized in the tables, we discuss the changes in the annual number of publications in the last part of this review. 

## 2. Terminology of SWNTs

**Figure 1:**
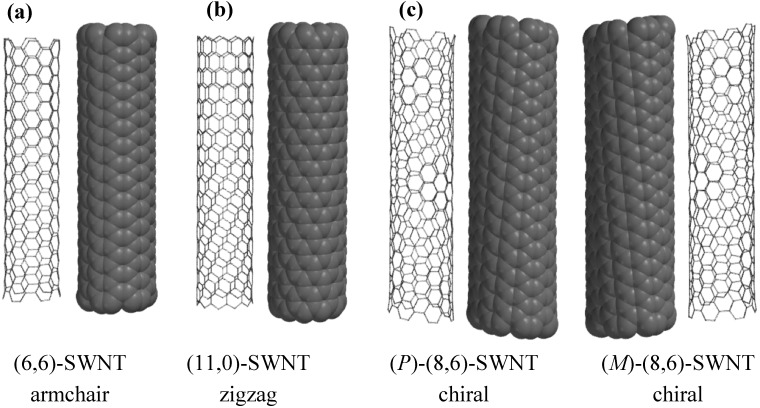
(a) Armchair, (b) zigzag, and (c) chiral SWNTs. *P* and *M* describing the handedness of CNTs are defined in Figure 2 and Figure 3.

**Figure 2:**
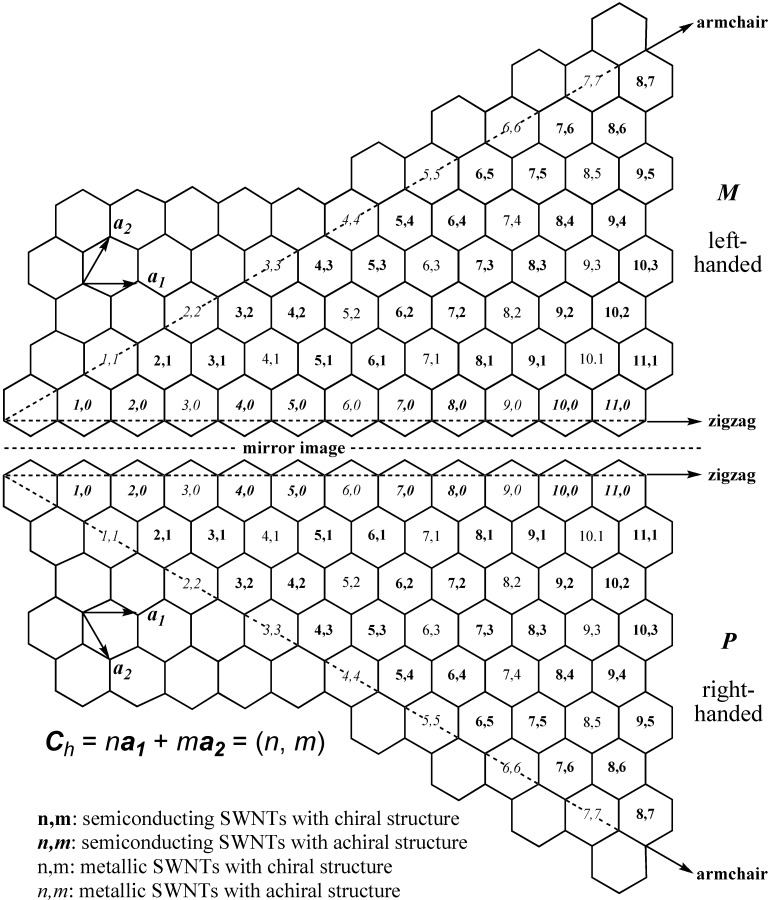
(*M*)- or (*P*)-(*n*, *m*)-SWNT defined by rolling up the graphene along the roll-up vector *C_h_* from the front to the back, so that the front and back sides become the outer and inner surfaces of the SWNT, respectively.

Before going into the main subject of this review, the terminology of SWNTs is briefly introduced in this section. The properties of SWNTs are determined by the alignment of the hexagons as shown in Figure 1. For their electronic properties, armchair SWNTs have metallic property, and zigzag and chiral ones are either metallic or semiconducting, depending on the roll-up index (see Figure 2). While armchair and zigzag are achiral, a chiral SWNT has a pair of helical isomers as shown in Figure 1 and Figure 2. Optically active SWNTs have been found by enrichment of either left- or right-handed structures [25,26,27,28,29,30].

Since a SWNT can be prepared by rolling up a graphene into a seamless cylinder, the structure is defined by a roll-up vector ***C****_h_* defined by two unit vectors ***a_1_*** and ***a_2_***; *C_h_* = *n**a_1_*** + *m**a_2_***, where *n* and *m* are integers and designated as the roll-up index (*n*, *m*) as shown in Figure 2 [31,32]. The (*n*, *m*) and ***C****_h_* have been referred to as “chiral” index (or simply “chirality”) and “chiral” vector, respectively. However, the meaning of the “chiral” is not consistent with the original meaning defined by the International Union of Pure and Applied Chemistry (IUPAC); that is, “the geometric property of a rigid object of being non-superposable on its mirror image” [33]. Since this terminology is confusing, as Strano pointed out in the article [34], an explicit nomenclature is required to define the structures of CNTs. In this review, the terms of “chiral” and “chirality” are only used according to the definition of IUPAC mentioned above, and the term of (*n*, *m*) is used for describing the roll-up index of SWNTs. 

**Figure 3:**
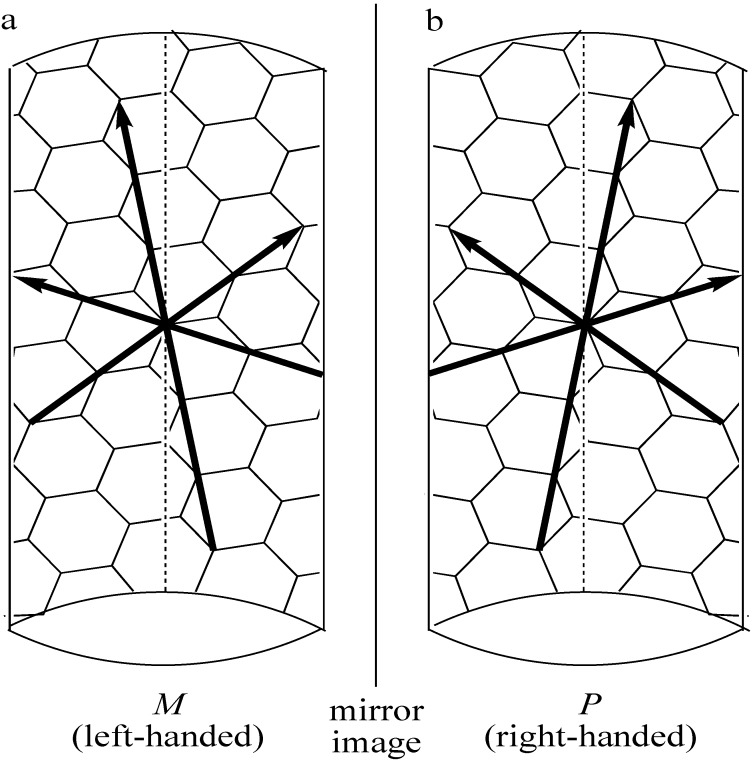
Definition of (a) *M* (left-handed) and (b) *P* (right-handed) chiral SWNTs. Three arrows and dashed line indicate zigzag lines and SWNT axis, respectively.

The handedness of chiral SWNTs is defined as *M* and *P* as follows. Every SWNT has three zigzag lines (Z lines) as indicated as solid arrows in Figure 3. These Z lines cannot be superposed on their mirror-image in the case of chiral SWNTs, while they can be superposed in zigzag and armchair types. When two of the three Z lines are rotated to the left and the third Z line to the right, the chiral SWNT is designated as *M* as shown in Figure 3a. Similarly, the chiral SWNT with two Z lines rotated to the right is *P* as shown in Figure 3b. This terminology is based on Z*R* and Z*L* in the literature [31]. In this review, we propose *M* and *P* stereodescriptors according to the IUPAC nomenclature.

## 3. Physical Methods for Separation of Carbon Nanotubes

### 3.1. Electrophoresis

Electrophoresis has been employed for separating various kinds of SWNTs, synthesized by laser vaporization (LV), arc discharge (AD), chemical vapor deposition (CVD) and HiPco (high pressure CO) process, according mainly to electrical property (M/S) together with length and diameter as summarized in Table 1 [35,36]. Charged biological macromolecules are commonly separated by electrophoresis using gel in an electric field. In order to process SWNTs in the gel, the SWNTs have to be individually dispersed with aid of surfactant such as sodium dodecylsulfonate (SDS), sodium cholate (SC) and sodium dodecylbenzenesulfonate (SDBS). The M/S separation using this method utilized different polarizable characters between them under an electric field. Dielectrophoresis technique was first used for the alignment and purification of SWNT bundles in isopropyl alcohol [37,38]. It was extended to separation of individually dispersed SWNTs.

**Table 1:** Separation of SWNTs by electrophoresis.

Reference	Separated object	SWNTs	Chemicals
[39,40]	M/S	HiPco, LV	SDS/D_2_O
[41]	M/S	LV	DMF
[42]	M/S	CVD	DMF
[43]	M/S	LV	SC/D_2_O
[44]	length, diameter	HiPco	SC/water
[45]	length	LV, HiPco	polyvinyl pyrrolidone (PVP)
[46]	length	AD	SDS/water
[47]	diameter	HiPco	SDS or PVP
[48]	M/S	HiPco	SDS/water
[49]	M/S, diameter	HiPco	SDBS/water
[50,51,52]	M/S	HiPco	SDS/water
[53,54,55]	M/S	LV, AD, HiPco	SDS/water
[56]	M/S	AD	SDS/water
[57]	M/S	HiPco	SDBS/D_2_O
[58,59]	M/S	HiPco, LV	Triton X-100, water
[60]	M/S	HiPco	SDS, cetyltrimethylammonium bromide
[61]	M/S	AD	Gum Arabic or PVP
[62]	diameter, length	HiPco	DNA or RNA

### 3.2. Centrifugation

This process is very powerful and versatile to separate SWNTs according to their electrical and structural properties such as M/S, diameter, length, (*n*, *m*) and handedness [63]. In 2005, Arnold and Hersam reported the first example of density gradient ultracentrifugation (DGU) for separation of the diameter of SWNTs [64]. They successfully applied the process to the separation of M/S electrical property, (*n*, *m*) structures and even handedness [25,65]. The methodology was extended to (*n*, *m*) separation and enantiomer sorting of HiPco SWNTs [30]. The papers reported so far are summarized in Table 2. SDS and/or SC have been commonly used as detergents to dissolve SWNTs. In addition, iodixanol is required as a gradient-induced agent to the aqueous medium. Organic system was employed in only one example in the presence of polymer and heavily halogenated benzene in organic solvent [66]. Quite recently, facile M/S separation has been realized by use of SDS and agarose gel [53,67].

**Table 2:** Separation of SWNTs by density gradient ultracentrifugation (DGU).

Reference	Separated object	SWNTs	Chemicals
[40,64,65,68,69,70,71,72,73,74,75,76]	M/S, diameter, (6,5), (7,5)	CoMoCAT, LV, HiPco, AD	SDS, SC/iodixanol in water, DNA/iodixanol in water
[77,78]	M/S	LV, HiPco, CoMoCAT	sodium deoxycholate (SDC)/ iodixanol in water
[25]	handedness, (6,4), (6,5)	CoMoCAT, AD	SC/iodixanol in water
[30]	handedness, (6,4), (6,5), (7,3), (9,1), (8,3), (9.2), (7,5), (8,4), (10,2), (7,6)	HiPco	SC, SDS/iodixanol in water
[66]	(7,5), (7,6), (10,5), (9,7)	LV, HiPco, CoMoCAT	fluorene-based polymer/chlorobenzene + tribromotoluene
[79]	M/S, diameter	HiPco	SDS, NaCl/iodixanol in water
[80]	M/S, diameter	AD	sodium taurodeoxycholic acid, SDS, SC/iodixanol in water
[81]	M/S	AD	SC, SDS/sucrose in water
[82]	(6,5)	Co-MCM-41	SC, SDS/iodixanol in water
[83]	diameter	HiPco	SDS, PVP/water
[84,85]	diameter	AD	SC/iodixanol in water
[86]	DWNTs	Mixture of SWNTs, DWNTs and MWNTs	SC/iodixanol in water
[87]	short length (about 7.5 nm)	HiPco	PL-PEG/iodixanol in water
[88]	length	CoMoCAT, HiPco, LV	SDC/iodixanol in water
[53,67]	M/S	LV, AD, HiPco	SDS/agarose gel
[89]	(*n*, *m*)	CoMoCAT, HiPco, LV	SC/iodixanol in water
[90]	length	CoMoCAT, HiPco, LV	SDC/iodixanol in water

## 4. Chemical Methods for Separation of Carbon Nanotubes

### 4.1. Chromatography

Chromatography was first employed for separation of CNTs by Duesberg and coworkers in 1998. In addition to removal of metallic impurities and amorphous carbon, length separation of SWNTs as well as MWNTs was accomplished by size exclusion chromatography (SEC) after dissolvation of CNTs in the presence of SDS [91,92,93]. In 2003, Zheng and coworkers separated DNA-wrapped SWNTs by ion-exchange chromatography (IEC) to realize M/S, length and diameter separations [94,95]. Quite recently, the IEC separation was extended to strict (*n*, *m*) separation by applying appropriate sequences of DNA, providing many kinds of pure (*n*, *m*) of SWNTs [96]. The separations of length and M/S by field flow fractionation (FFF) and optical trapping, respectively, are incorporated in this section, as shown in Table 3 [97,98,99,100,101,102,103].

**Table 3:** Separation of SWNTs by chromatography.

Reference	Separated object	SWNTs	Chemicals
[104]	(8, 4)	FeRu-CVD	DNA, IEC
[105]	(6, 4), (9, 1), (6, 5)	CoMoCAT	DNA, SEC + IEC
[96,106]	(*n*, *m*)	HiPco	DNA, IEC
[94,95,107,108,109]	M/S, diameter, length	HiPco	DNA, IEC
[110]	(6, 5)	CoMoCAT	DNA, IEC
[111]	M/S, diameter, (*n*, *m*)	HiPco	DNA, SEC + IEC
[112,113,114]	length		DNA, SEC
[115]	length	HiPco	octadecylamine/THF, GPC
[91,92,93,116]	length	LV, AD, MWNTs	SDS, SEC
[117]	length	LV	Triton X-100, SEC
[44,118]	length, diameter	HiPco, LV	SC, SEC
[119]	length	CoMoCAT, HiPco, LV, AD	DNA, SEC
[120]	M/S	HiPco functionalized with t-aryl groups group	SDS/o-dichlorobenzene, silica gel chromatography
[121]	M/S	HiPco	SDS/agarose gel beads
[40]	M/S	LV	SDS, SC/SEC
[97]^a^	length	LV	Triton X-100, water
[98]^a^	length	AD (SWNTs), CVD (MWNTs)	SDS, water
[99]^a^	length	AD	Triton X-100, water
[100]^a^	length	CNT (Carbolex)	Triton X-100, water
[101]^a^	length	CoMoCAT, HiPco, LV, AD	DNA, water
[102]^a^	length	functionalized MWNTs	water (pH = 10)
[103]^b^	M/S	HiPco	DNA, water

^a^ FFF: Field-flow fractionation, ^b^ Optical trapping.

### 4.2. Selective Solubilization

Although SWNTs are not solubilized in any solvents, they can be dissolved into solvent in the presence of a solubilizing agent [12,13,14,15,16,17,122]. If the agent can recognize the electrical property and/or structural character of SWNTs, separation of SWNTs can be realized through the extraction [123]. The solubilizing agents reported so far include simple molecule such as alkylamine for M/S separation [124,125,126,127,128,129,130], polymers such as fluorene-based ones for extracting specific (*n*, *m*) structure [66,89,131,132,133], poly(phenylenevinylene) [134,135,136,137,138] and designed host molecules such as tweezer-type ones for discrimination of diameter, (*n*, *m*) and handedness [26,28,29,34]. Although the SWNTs have been optically resolved by selective solubilization with chiral nanotweezers [26,27,28,29] and DGU with chiral detergent [25,30], the absolute configuration of the resolved SWNTs were determined only by theoretical calculations [139]. Therefore, experimental evidence is required to assign the handedness as Weisman pointed out in his recent paper [30]. The papers of selective solubilization are summarized in Table 4. 

**Table 4:** Separation of SWNTs by selective solubilization.

Reference	Separated object	SWNTs	Chemicals
[53,140]	M/S	LV	SDS/water
[124,125,126,127]	M/S	LV, HiPco	octadecylamine, octadecylamine/THF
[128,129,130]	M/S (87% M)	HiPco, CoMoCAT	octylamine/THF
[141]	M/S	AD	amine- or phenyl-terminated SiO_2_
[142,143]	M/S	LV, 1.1-1.6 nm	bromine, triton X-100/water
[130,144,145]	M/S	AD	porphyrin/CHCl_3_, pyrene/THF
[146,147]	(8, 6) 85%	HiPco	flavin mononucleotide/D_2_O
[131]	(7, 5)	Co-MCM-41	fluorene-based polymers/toluene
[132,133]	(7, 5), (8, 6), (10, 5)	CoMoCAT, HiPco	fluorene-based polymers/toluene, xylene, THF, chloroform
[148,149]	(8, 6), (7, 6) diameter	HiPco	pentacene-quaterrylene- and naphthopentaphene-based amphiphiles, SDS/water
[66]	(7, 5), (7, 6), (10, 5), (9, 7)	LV, HiPco, CoMoCAT	fluorene-based polymer/chlorobenzene + tribromotoluene
[89]	(*n*, *m*)	LV, HiPco, CoMoCAT	fluorene-based polymer/toluene
[134,135,136,137]	diameter, M/S	AD, HiPco	poly(phenylenevinylene)/toluene
[138]	(11,6), (11,7), (12,6)	HiPco	poly(phenylenevinylene)/THF
[150,151]	diameter	HiPco	reversible cyclic peptide/water
[152]	diameter	AD	η-cyclodextrin/D_2_O
[153]	diameter	SWNTs	pentacene-based molecular tweezers/toluene
[154]	M/S	AD	potassium salt of coronene tetracarboxylic acid/water
[155]	diameter	HiPco	chitosan polymer/water
[156]	diameter	HiPco	porphyrinic polypeptides/DMF
[157]	diameter	HiPco	ruthenium metallodendrimer/DMF
[27]	helicity, diameter	CoMoCAT	chiral monoporphyrin/methanol
[26,28,29,34]	helicity, (n, m)	CoMoCAT	chiral nanotweezers/metanol
[158,159]	diameter	HiPco, AD	diamine-terminated oligomeric poly(ethylene glycol)/water
[160]	length	HiPco	tetraoctylammonium bromide/ethyl acetate or toluene
[161]	(8, 4), diameter	CoMoCAT, HiPco	heparin/water heparin, SDBS/water
[162]	M/S	CoMoCAT	DNA/water
[130,163]	M/S, diameter	HiPco	pyrene derivative/water
[164]	diameter	CoMoCAT, HiPco	pyrene derivative/water
[165]	diameter	HiPco	SDS, SDBS or SC in water
[166]	diameter	HiPco	ClSO_3_H/CH_3_SO_3_H

### 4.3. Selective Reaction

Most of the selective reactions for SWNT separation [123,167] are classified into the following three types of chemical and physical processes; selective oxidation in the presence of H_2_O_2_ [168,169,170,171,172], OsO_4_ [173], H_2_SO_4_/HNO_3_ [174,175,176,177], HNO_3_ [178], ozone [179,180], AuCl_4_^–^ [181], NaClO_x_ [182] and air (high temperature) [183,184] for M/S, diameter and (*n*, *m*) selections, selective reaction with nitronium ion [185,186], NO_2_ [187], carbene [188,189], diazonium salt [58,109,190,191,192,193,194,195,196,197,198,199,200,201,202], fluorine [203], triethylsilane [204], fluorinated olefin [205], SO_3_ [206], RLi and RMgX [207], and azomethine ylide [208] for M/S separation, selective break-down of either metallic or semiconducting SWNTs by use of electricity [209,210,211], plasma [212,213], laser [214,215], microwave [174,216,217] and Xe-lamp [218], and electrochemical doping with Li [219] and cations [220,221] to specific (*n*, *m*) and diameter. The literature is summarized in Table 5.

**Table 5:** Separation of SWNTs by selective reaction.

Reference	Separated object	SWNTs	Chemicals
[130,168]	M/S	HiPco	H_2_O_2/_water at 90 °C
[169]	diameter, (*n*, *m*)	HiPco	air at 450 °C, H_2_O_2/_water at 90 °C
[170]	diameter	HiPco	H_2_O_2_, light irradiation
[171]	(*n*, *m*)	HiPco, CoMoCAT	H_2_O_2_, SC or SC/SDS in D_2_O
[172]	diameter	HiPco	H_2_O_2_ + light
[173]	M/S	HiPco	OsO_4_, UV
[182]	M/S	AD	NaClO_x_/1-methyl-2-pyrrolidone
[174]	M/S, diameter	HiPco	acid mixture (H_2_SO_4/_HNO_3_) under microwave irradiation
[175]	M/S	HiPco	acid mixture (H_2_SO_4/_HNO_3_)
[176,177]	diameter	HiPco	acid mixture (H_2_SO_4/_HNO_3_) at 35–55 °C under sonication
[178]	M/S	HiPco	HNO_3_ at 135 °C
[179,180]	diameter	HiPco	ozone in methanol at −78 °C
[181]	(6, 5)	HiPco	AuCl_4_^–^, SC/water
[183]	diameter	LV	air at 350–550 °C, HNO_3_ at 120 °C
[184]		HiPco	air at 460–620 °C
[185,186]	M/S	HiPco	NO_2_SbF_6_ or NO_2_BF_4_ in tetramethylene sulfone/chloroform
[187]	M/S, diameter	SWNTs	NO_2_
[188,189]	M/S	HiPco	dichlorocarbene in dichlorobenzene
[190,194,197,198,199]	M/S	HiPco	diazonium salt/water
[191]	M/S	AD	diazonium salt of 4-heptadecafluorooctyl-aniline/perfluorohexane
[192,195]	M/S	HiPco, AD	4-nitrobenzenediazonium salt in DMF, 4-aminobenzylamine
[193,200]	M/S	CVD	4-bromobenzenediazonium tetrafluoroborate in water
[58,109,201]	M/S	HiPco	4-hydroxybenzenediazonium salt
[196]	M/S	HiPco	SC, 4-dodecyloxybenzenediazonium tetrafluoroborate in water
[202]	M/S, diameter	HiPco	SDS, 4-chloro- and 4-nitrophenyldiazonium salts in water
[203]	M/S, diameter	HiPco	fluorine gas
[204]	M/S	HiPco	triethylsilane at room temperature
[205]	M/S	HiPco	perfluoro 2-(fluorosulfonylethoxy)propyl vinyl ether at 215 °C
[206]	M/S	HiPco	SO_3_ at 400 °C
[130,208]	M/S	HiPco	azomethine ylide/THF at 65 °C
[207]	M/S, diameter	HiPco	RLi, RMgX in cyclohexane
[209,210,211]	M/S	MWNTs, SWNTs	current-induced electrical breakdown
[212]	M/S	CVD	methane plasma at 400 °C/annealing at 600 °C
[213]	M/S	AD	hydrogen plasma
[214]	(*n*, *m*)	CVD	laser irradiation
[215]	M/S	CoMoCAT, HiPco, LV	laser irradiation
[218]	M/S	Fe-catalyzed CVD	Xe lamp
[216]		CoMoCAT	microwave irradiation
[174,217]	M/S, diameter	HiPco	microwave irradiation
[219]	diameter	HiPco	Li at 473 °C
[220]	(*n*, *m*)	HiPco	SDS in D_2_O/salt (NaCl, MgSO_4_, ErCl_3_)
[221]	diameter	HiPco	LiClO_4_, (CH_3_)_4_NBF_4_, n-Bu_4_NClO_4_, n-Oct_4_NClO_4_, ionic liquid in CH_3_CN
[222]	M/S	AD	aromatic or aliphatic solvent with electron-withdrawing or -donating groups
[223]	(*n*, *m*)	HiPco	TCNQ, TFTCNQ^a^, mordant yellow 10 and AB^b^
[224]	M/S	(10, 0), (6, 6)	naphthalene, anthracene, TCNQ and DDQ

^a^ 2, 3, 5, 6-tetrafluoro-7, 7, 8, 8-tetracyanoquinodimethane, ^b^ 4-amino-1, 1-azobenzene-3, 4-disulfonic acid.

## 5. Concluding Remarks

**Figure 4:**
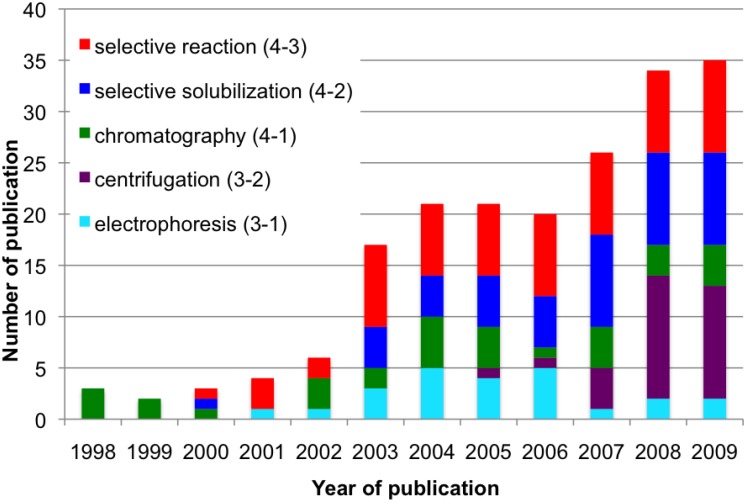
Annual change in number of publications in the following five methods; electrophoresis (3-1), centrifugation (3-2), chromatography (4-1), selective solubilization (4-2) and selective reaction (4-3).

**Figure 5:**
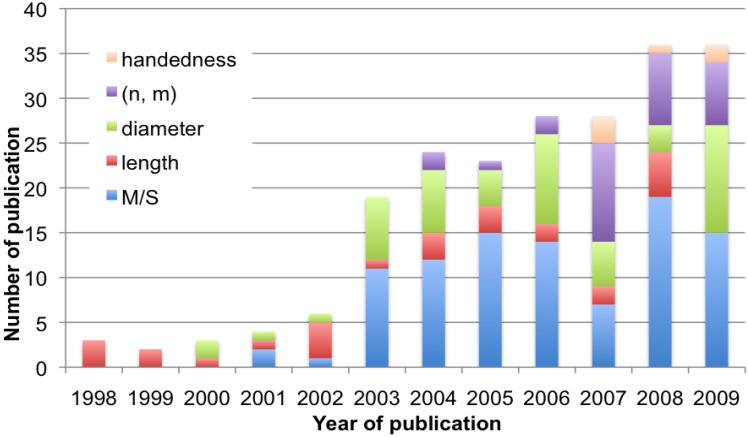
Annual change in number of publications in the following targeted objects to be separated; M/S, length, diameter, (*n*, *m*) and handedness.

**Figure 6:**
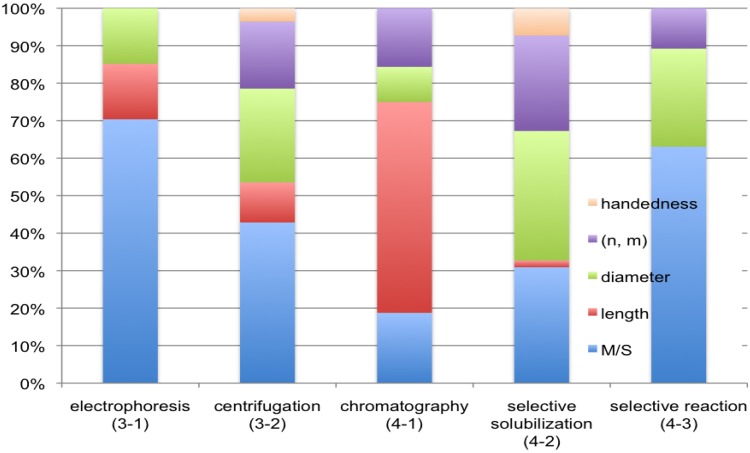
Rate in targeted object of SWNTs to be separated in the following five methods; electrophoresis (3-1), centrifugation (3-2), chromatography (4-1), selective solubilization (4-2) and selective reaction (4-3).

Separation of SWNTs has been described in this review. The related papers reported so far are classified into the following five methods; electrophoresis (Chapter 3.1), centrifugation (3.2), chromatography (4.1), selective solubilization (4.2) and selective reaction (4.3). All the data, summarized in Table 1, Table 2, Table 3, Table 4 and Table 5, will be analyzed on the basis of Figure 4, Figure 5 and Figure 6.

Changes in the annual number of publications from 1998 to 2009 are shown in Figure 4. The first few reports, published in 1998–1999, dealt with length separation of CNTs by chromatography and the related technique. CNTs were solubilized by use of sonication in the presence of surfactant in 1997 [225,226], making the SEC [91,92,93] and FFF [97] separations possible. The first papers on selective solubilization by polymer wrapping [134] and electrophoresis of polymer-wrapped SWNTs [45] were published in 2000 and 2001, respectively. The annual number of publications is less than 10 until 2002. During this period, about 60% of the papers focus on length separation, while investigations started on diameter and M/S separations (Figure 5). Noteworthy is that the first M/S separation was accomplished by electrical breakdown in 2001 [209,210]. In 2003, the number of papers suddenly leapt to more than 15 papers (Figure 4). This is probably because individualization of SWNTs in aqueous solution and the spectroscopic analysis were accomplished in 2002 by Smalley and Weisman [122,227]. The findings greatly facilitate more precise evaluation of the distribution of diameters and (*n*, *m*) structures of the SWNTs before and after separation. Novel methods of selective reaction of carbenes and diazonium salts with metallic SWNTs were devised in 2003 [188,189,190,194], also increasing the number of publications in 2003 (Figure 4). Especially, publications related to M/S and diameter separations increased remarkably in 2003, as shown in Figure 5. In 2005 and 2006, the first papers of DGU were published by Hersam and coworkers [64,65], causing a sudden increase of the number of papers to more than 25 in 2007 (Figure 4). Fluorene-based polymers also found to extract the specific structure in 2007 [132]. These novel methodologies realized precise (*n*, *m*) separation and enhanced the number of publications related to the (*n*, *m*) separation shown in Figure 5. In the same year, optically active SWNTs were obtained for the first time by separating the handedness of chiral nanotubes [29]. Conclusively, the changes in annual number of publications shown in Figure 4 and Figure 5 indicate clearly that new findings on evaluation and separation of SWNTs stimulated the researchers in this field to increase the number of publications. The annual number of publications in this field is still increasing as shown in Figure 4 and Figure 5. Several papers have already appeared in the beginning of 2010 [30,154,161,164].

Figure 6 summarizes the rates of the separated objects in SWNTs in each of the five separation methods; electrophoresis, centrifugation, chromatography, selective solubilization and selective reaction. The rate is different among these methods as shown in the figure; for example, electrophoresis and selective reaction have been employed mainly for M/S separation, while length separation has been carried out mostly by chromatography. Centrifugation has been used for separating SWNTs according to a variety of structural and electrical features including (*n*, *m*) and even handedness. However, the method is not considered to be suitable for large-scale separation. For M/S separation, scalable methods have been reported recently by Kataura and coworkers [140]. A more facile method to obtain specific (*n*, *m*) in large quantities is awaited in view of electrical and optical applications of SWNTs. 
